# Safeguarding genomic imprints in naive human pluripotency

**DOI:** 10.1016/j.stemcr.2025.102475

**Published:** 2025-04-17

**Authors:** Alejandro De Los Angeles

**Affiliations:** 1 Harvard Medical School, Boston, Massachusetts, USA; 2 Beth Israel Deaconess Medical Center, Boston, Massachusetts, USA; 3 Massachusetts Institute of Technology, Cambridge, Massachusetts, USA; 4 Broad Institute of MIT and Harvard, Cambridge, Massachusetts, USA

## Abstract

Naive human pluripotent stem cells (hPSCs) closely mirror the pre-implantation epiblast but risk imprint erosion under strong MEK/ERK inhibition, jeopardizing disease modeling and regenerative applications. In *Stem Cell Reports*, Fischer et al. show that partial MEK/ERK inhibition plus ZFP57 overexpression crucially preserves parent-of-origin DNA methylation, thereby offering more faithful and stable naive hPSC models.

## Introduction

**Figure 1:**
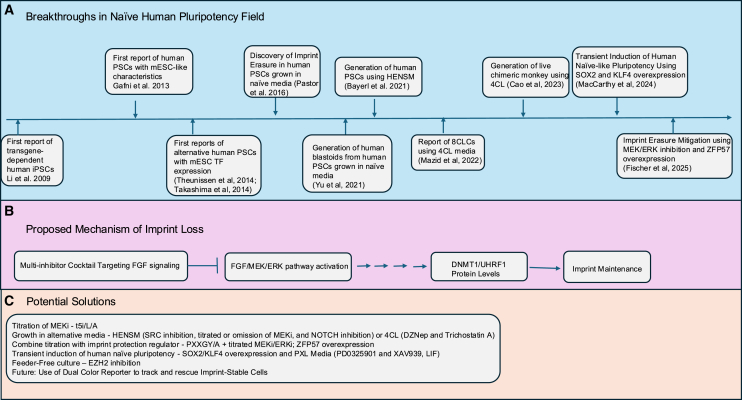
Breakthroughs in naive hPSC derivation, mechanisms of MEK/ERK-driven imprint loss, and strategies for maintaining stable imprints (A) Timeline of naive hPSC breakthroughs, highlighting discovery of different cell types, media compositions, different characterization, and functional assays. (B) Flow diagram showing how strong MEK/ERK inhibition can lead to imprint loss via direct or indirect inhibition of DNMT1 and/or UHRF1, resulting in biallelic expression (LOI). (C) Bottom: potential solutions, including partial MEK/ERK inhibition, ZFP57 overexpression, and other modifiers (e.g., PRC2/NOTCH inhibitors). The use of a dual-color reporter approach can facilitate the identification of conditions that promote the isolation of imprint-stable cells.

Over the past decade, researchers have sought to derive and stabilize naive human pluripotent stem cells (hPSCs)—*in vitro* counterparts to the pre-implantation epiblast. Protocols that convert “primed” hPSCs (similar to the post-implantation epiblast) into a “naive” state have proliferated (Figure 1). Early work using naive human stem cell medium combined FGF stimulation and MEK inhibition to confer key features of murine embryonic stem cells (mESCs) upon hPSCs (Gafni et al., 2013). Subsequent media, such as 5i/L/A and t2iLGö, further promoted naive-like gene expression including induction of transcription factors associated with murine naive pluripotency (Takashima et al., 2014; Theunissen et al., 2014). However, these latter culture conditions, marked by genome-wide DNA hypomethylation, often caused genetic and epigenetic instability, including karyotypic abnormalities and erosion of DNA methylation at imprinted loci (Pastor et al., 2016).

Genomic imprinting involves parent-of-origin-specific DNA methylation marks that govern monoallelic expression of genes like SNRPN, IGF2, and H19—critical regulators of embryonic growth. Normally, these imprints remain intact through early embryonic development until germline reprogramming. Yet in many naive induction protocols, imprint erosion has emerged as a persistent challenge. Once imprints are lost, returning cells to primed conditions does not restore them, raising concerns for disease modeling, embryo-like structure generation, and regenerative therapies. Several groups have sought to address this problem by modifying naive culture conditions. Novel media such as HENSM or 4CL reported improvements in imprint preservation (Bayerl et al., 2021; Mazid et al., 2022), but imprint instability remains a common roadblock in other naive induction protocols (Pastor et al., 2016).

In this issue of *Stem Cell Reports*, Fischer and colleagues directly tackle imprint erosion using a dual-color fluorescent reporter at the SNRPN locus in naive hPSCs (Fischer et al., 2025). Their single-cell measurements reveal when and how erosion occurs, and then show that lowering MEK/ERK inhibition and overexpressing the regulator ZFP57 can partially preserve imprints. Their findings underscore that naive induction must be carefully fine-tuned to prevent loss of essential epigenetic marks.

## Imprint erosion in naive hPSCs

In mice, embryonic stem cells (ESCs) are derived from the pre-implantation epiblast and can be stabilized in 2i/LIF (MEK and GSK3 inhibition plus LIF) (Ying et al., 2008). Despite this powerful approach, extended passage of mESCs in 2i can also erode certain imprints (Choi et al., 2017). When comparable strategies were applied to hPSCs—e.g., 5i/L/A and t2iLGö—they triggered marked global hypomethylation, including at many imprint control regions (ICRs) such as H19/IGF2 and SNRPN (Pastor et al., 2016). In some lines, 5-methylcytosine levels dropped by 50% or more.

A recent study by Keshet and Benvenisty demonstrated that imprinting aberrations in naive hPSCs are often locus specific and linked to paternally methylated imprinted genes (Keshet and Benvenisty, 2021). Their data suggest that reduced FGF signaling correlates with more severe imprint erosion, possibly explaining why certain induction protocols produce heavier imprint damage.

In certain naive pluripotent stem cell (PSC) culture conditions, MEK inhibition—central to activating naive-specific transcription—appears to accelerate passive DNA demethylation by directly or indirectly causing the reduction of protein expression levels of DNMT1 and UHRF1, inhibiting maintenance of DNA methylation (Pastor et al., 2016). While this widespread demethylation can eliminate unwanted epigenetic “memory” from primed states, it also destabilizes valuable imprinting marks, and once these marks are gone, reintroducing them has proven difficult.

## Real-time tracking and mitigation

Fischer and colleagues engineered a dual-fluorescent reporter at the SNRPN locus, crucial in the Prader-Willi/Angelman region on chromosome 15. Under normal imprinting, only the paternal SNRPN allele is expressed (driving mRuby3), while the maternal allele (tagged with EGFP) remains silent. During naive induction, if the maternal allele loses its imprint, EGFP expression emerges—giving a direct “live” readout of imprint erosion at single-cell resolution.

By mapping fluorescence in real time, Fischer et al. found that strong MEK/ERK inhibition can rapidly induce biallelic expression of SNRPN. Once cells become biallelic, reverting them to primed culture does not restore monoallelic expression. Moreover, not all cells are equally affected; some subpopulations preserve imprinting.

To prevent erosion, Fischer et al. tested two main strategies:

(1) Titrating MEK/ERK inhibition: reducing inhibitor concentration (relative to “full-strength” protocols) delayed and decreased imprint loss, though the overall efficiency of naive induction dropped somewhat (Di Stefano et al., 2018; Bayerl et al., 2021).
(2) Overexpressing ZFP57: as a key KRAB zinc-finger protein, ZFP57 binds methylated ICRs. Overexpressing it during reprogramming partially protected imprint stability, and combining it with milder MEK/ERK inhibition produced an even greater protective effect.

Although these experiments provide valuable insight, it is worth noting that certain observations—such as the rescue of imprint stability—may be partly conditioned by the specific PXGGY/A culture environment used in the study by Fischer et al. (Khan et al., 2021). In fact, while the authors primarily relied on PXGGY/A, they confirmed key findings (including ZFP57’s protective effect) in 5i/L/A as well, suggesting that imprint rescue is not exclusively tied to one medium. Future studies will be needed to confirm whether a similar imprint-preserving effect occurs under other naive media or conditions.

This work offers a blueprint for mitigating imprint erosion: fine-tune exogenous signals and bolster imprint regulators. Their single-cell readouts provide a means to isolate subpopulations that maintain correct imprinting, which is invaluable for downstream applications.

## Technological innovations driving the field

### Single-cell epigenomics and reporters

By pairing fluorescent reporters with single-cell sequencing, researchers can track loss of imprinting (LOI) in real time and dissect the subpopulations most vulnerable to demethylation. Tools like Fischer et al.’s SNRPN reporter enable continuous monitoring of imprint status alongside manipulations of signaling pathways or epigenetic regulators.

### Refined and optimized culture systems

Early naive media relied heavily on MEK/GSK3 inhibitors (2i) or alternative blockade of FGF signaling (5i/L/A), often causing global hypomethylation. The field is moving toward more subtle modifications—e.g., reduced inhibitor doses, alternate perturbations such as CDK8/19 blockade, and partial FGF pathway manipulation—to activate naive transcription programs without stripping away vital imprint marks.

### Gene editing and overexpression

CRISPR-Cas9 facilitates precise insertion of reporters and the stable overexpression of protective factors like ZFP57 or ZNF445. Imprint maintenance likely involves several players, including other KRAB zinc-finger proteins and maternal-effect genes. Systematic screening of these candidates may uncover further ways to shield imprints during reprogramming.

## Biological and translational relevance

Naive hPSCs with properly maintained imprints promise major benefits for basic science and potential therapies. Many labs aim to use naive cells to

(1) model pre- and peri-implantation stages *in vitro*,
(2) derive trophoblast or germline lineages, and
(3) create embryo-like structures (blastoids) or integrated embryo models for studying early developmental events.

However, if naive hPSCs systematically lose or biallelically express imprinted genes like IGF2, H19, or SNRPN, these disease or developmental models become unreliable.

From a therapeutic standpoint, imprint dysregulation is implicated in syndromes such as Prader-Willi, Angelman, and Beckwith-Wiedemann and can affect cell proliferation or tumorigenicity. Fischer et al.’s demonstration of imprint protection thus brings the field closer to safe, stable naive hPSCs, enabling faithful disease modeling and potential cell-based interventions.

## Near-future steps

### Combining partial signaling inhibition with additional regulators

ZFP57 is only one piece of the puzzle; other factors (e.g., ZNF445, DPPA3/STELLA, or certain maternal NLRP proteins) may work synergistically to protect imprints. Exploring these candidates in parallel with partial MEK inhibition could further enhance imprint stability.

### Refinement of feeder-free systems

Certain media (e.g., HENSM) combine MEK, tankyrase, and PKC blockade with SRC inhibition and NOTCH inhibition and specialized substrates (Bayerl et al., 2021). A more recent study identified PRC2 inhibition as a perturbation that stabilizes feeder-free growth of human naive PSCs (Huang et al., 2025). It remains to be seen how these modifications affect multiple imprinted loci beyond SNRPN. Additional systematic testing might identify culture conditions that minimize LOI across the board.

### Multiplexed reporters for global imprint surveillance

Fischer et al. focused on the SNRPN domain, but other loci like H19/IGF2 could also be tagged. High-throughput screens could then reveal small molecules or culture modifications that protect several imprinted regions at once, while sustaining naive transcriptional programs.

### Assessing long-term stability and differentiation

Even if imprint loss is reduced early on, it is unclear whether partial MEK inhibition and ZFP57 overexpression preserve these marks through extended culture or subsequent differentiation. Evaluating stability over clinically relevant times and multiple passages is essential.

### Direct conversion and early intervention

Many naive lines are derived from primed PSCs, a process that may expose cells to prolonged demethylating conditions. Direct reprogramming of somatic cells into naive iPSCs—incorporating ZFP57 or other imprint guardians early—could minimize the time window in which imprints are vulnerable.

### Pursuing transient induction strategies

Some researchers are testing whether a brief “pulse” of naive conditions, such as SOX2 and KLF4 overexpression, is enough to reset epigenetic barriers without long-term instability (MacCarthy et al., 2024). Transient induction could remove undesirable primed-state memory yet avoid deep imprint erosion. Future studies must confirm that such short exposures preserve hallmark naive features (e.g., trophoblast and blastoid competence) and evaluate whether protective factors (e.g., ZFP57 or STELLA) should be introduced during these pulses.

### Toward stem cell-derived embryo models

As teams strive to recreate peri-implantation events using blastoids or other stem cell-derived embryo models, ensuring correct imprinting from the start is crucial. Proper dosage of paternally and maternally expressed genes is critical for normal placental development, early embryonic growth, and accurate disease phenotypes *in vitro*.

## Conclusion

By implementing a sophisticated dual-fluorescent SNRPN reporter, Fischer and colleagues deliver critical insights into how imprint erosion unfolds—and can be curtailed—during naive hPSC induction. Their findings confirm that once a locus loses its imprint, reverting to primed culture typically cannot restore correct methylation patterns. This underscores the imperative to prevent erosion from the outset.

Equipped with these lessons, future studies can refine naive culture protocols to preserve imprint stability, whether by modulating MEK/ERK inhibition, introducing imprint regulators, or adopting transient induction strategies. Mastering imprint maintenance in naive hPSCs promises more faithful models of early human development and safer avenues toward regenerative therapies.

## Acknowledgments

I am indebted to the mentors and colleagues who generously shared their insights on pluripotency and reprogramming, shaping my expertise and fueling this work. Their guidance has been invaluable in bringing this research to fruition.

## Declaration of interests

The author declares no competing interests.

## Declaration of generative AI and AI-assisted technologies in the writing process

During the preparation of this work, the author used a large language model (ChatGPT) in order to refine language and grammar. After using this tool/service, the author reviewed and edited the content as needed and assumes full responsibility for all content.
